# Ambipolar surface conduction in oxygen sub-stoichiometric molybdenum oxide films

**DOI:** 10.1038/s41598-023-48060-1

**Published:** 2023-11-30

**Authors:** Anastasia Soultati, Konstantinos Aidinis, Alexander Chroneos, Maria Vasilopoulou, Dimitris Davazoglou

**Affiliations:** 1 Institute of Nanoscience and Nanotechnology, NCSR “Demokritos”, POB 60228, 153 10 Agia Paraskevi, Attiki Greece; 2 Department of Electrical and Computer Engineering, Ajman University, P.O. Box 346, Ajman, United Arab Emirates; 3 Center of Medical and Bio-Allied Health Sciences Research, Ajman, United Arab Emirates; 4 Department of Electrical and Computer Engineering, University of Thessaly, 38221 Volos, Greece; 5 Department of Materials, Imperial College, London, SW7 2AZ UK

**Keywords:** Materials science, Materials for energy and catalysis

## Abstract

The surface electric conduction in amorphous and crystallized molybdenum oxide films was studied as a function of electronic structure by current–voltage and simultaneous spectroscopic ellipsometry measurements on structures of the kind Al/Molybdenum oxide (MoO_x_)/Al, at temperatures up to 400 °C and in ambient air. At room temperature, both amorphous and crystalline MoO_x_ samples were found to be sub-stoichiometric in oxygen. The random distribution of oxygen vacancies and the imperfect atomic ordering induced the creation of an intermediate band (IB) located near the valence band and of individual electronic gap states. At temperatures below 300 °C, the conduction was found to exhibit ambipolar character in which electrons and holes participated, the former moving in the conduction band and the latter in the IB and though gap states. Above 300 °C, due to samples gradual oxidation and improvement of atomic ordering (samples crystallization), the density of states in the IB and the gap gradually decreased. The above in their turn resulted in the gradual suppression of the ambipolar character of the conduction, which at 400 °C was completely suppressed and became similar to that of ordinary n-type semiconductor. The above phenomena were found to be reversible, so as the semiconducting MoO_x_ samples were returning to room temperature the ambipolarity of the conduction was gradually re-appearing giving rise to an unusual phenomenon of “metallic” temperature variation of electrical resistance when electrons were injected.

## Introduction

Metal oxide films form a significant category of materials used in a number of applications including electrochromics^1^, gas sensing^2^, hybrid organic–inorganic light emitting diodes^3–5^ and solar cells^6,7^ silicon-based solar cells^8^, etc. Many of these oxides when fully stoichiometric in oxygen and perfectly crystallized are semiconductors exhibiting band gaps of the order of 3 eV, therefore are transparent within the visible part of the spectrum. However, deviations from full stoichiometry, imperfect atomic ordering and eventually doping when present in them alter their electronic structure causing the creation of individual states and even of bands within their band gap (intermediate bands, IBs)^9^. Electronic transitions between gap states and IB and the conduction band cause light absorption at energies below the band gap, so in many instances metal oxide films appear colored. The presence of gap states endows metallic oxides with very interesting physical–chemical properties^10^ the exploitation of which leads to the applications listed above. So, by controlling the exact oxygen stoichiometry and atomic ordering one may tailor the electronic structure of these materials, therefore their physical–chemical properties and design novel applications. Similarly to most semiconductors of interest, the electronic structure of metallic oxides near the band gap can be probed by studying their optical properties.

Except of the chemical affinity of the metal towards oxygen, two major factors influence the oxygen stoichiometry of metal oxides: the chemical composition and the temperature of the environment. This statement is easily understandable by simple thermodynamic considerations: At absolute zero it is theoretically possible for a lattice to exhibit no defects at all but as the temperature rises above zero, as foreseen by the Schottky–Wagner theory^11^, point defects form due to the migration of oxygen and metallic ions at interstitial or substitutional sites and on the surface, thus creating metallic and oxygen vacancies. The populations of thermally generated metallic and oxygen vacancies increase following Arrhenius-like dependences with activation energies generally different for the two kinds of ions and which generally vary with temperature and time. During atomic migration within the lattice three possible events may occur: (i) an oxygen ion meets a metallic ion; in this case the stoichiometry is not affected. (ii) Two metallic ions meet and form a chemical bond by sharing (fully or partially) their valence electrons. In this case the oxygen stoichiometry decreases because now two (at least) M–O bonds are eliminated to form one M–M bond (strong or loose) and the two oxygen ions remain loosely bonded in the lattice (if at all) and easily migrating towards the surface. (iii) Two oxygen ions meet and form an oxygen molecule, which is volatile and may escape easily out of the lattice. The last two processes are accelerated for metallic oxide films prepared under vacuum or during measurements in deep vacuum. Of the three described mechanisms, the last two lead to oxygen sub-stoichiometry and apply universally for all metallic oxides since they are dictated by thermodynamics. On the other hand, if a metallic oxide is found in an oxidizing chemical environment (e.g., ambient air) and (generally) above room temperature, oxidation may occur. The process of oxidation is related to microscopic processes such as the formation of point and extended defects, their migration within the lattice, the oxygen diffusion, etc., which in turn depend on the structure and the morphology of the lattice and the chemical affinity of the metal towards oxygen. The overall oxidation is also thermally activated and can be described with an Arrhenius-like law, so at some specific temperature ranges the oxidation is possible to recompense the thermodynamically favored sub-stoichiometry, thus resulting in films nearly or fully stoichiometric in oxygen.

In the case of deposited metal oxide thin films in which, with the exception of epitaxial ones, by definition the atomic arrangement is far from perfect, except of thermodynamics also the deposition method and the particular deposition conditions influence significantly the exact oxygen stoichiometry and atomic ordering thus providing additional degrees of freedom for the design of materials with properties tailored for specific applications. Dependent on the deposition method and conditions, point and extended defects, grain boundaries, voids, features with dimensions in the nano-scale, etc. may be formed, which except from oxygen stoichiometry, also affect the morphology of the film. The above features determine the short- and the long-range atomic order (SRO and LRO, respectively) in films, which in turn determine their electronic structure^12^ and consequently their physical properties.

In a previous work^9^, it was shown that while stoichiometric amorphous molybdenum oxide films exhibit a band gap of 3.2 eV free of electronic states, the oxygen sub-stoichiometry leads to the formation of electronic states within the gap and the formation of an IB centered approximately 2 eV below the edge of the CB exhibiting an extended tail towards the CB. Electronic transitions between the IB and the CB cause light absorption at energies below the band gap, which leads to the characteristic blue coloration of sub-stoichiometric molybdenum oxide films.

In this work the surface electric conduction in molybdenum oxide films at temperatures ranging from 25 to 400 °C with increments of 10 °C and in ambient air in relation to their electronic structure is investigated. The study is based on current–voltage (I–V) and spectroscopic ellipsometry (SE) measurements performed simultaneously at every temperature. The specific temperature range was chosen because such temperatures are commonly used during operation of devices based on metallic oxides (e.g., resistance gas sensors^2^), or during processing to fabricate various devices using molybdenum oxide films^3–8^. The study started with sub-stoichiometric in oxygen (MoO_x_, x = 2.7), highly disordered (amorphous) and porous^7,9^ molybdenum oxide films exhibiting deep blue coloration, which were attributed to the presence of the IB and of individual electronic states within the band gap. Due to this particular electronic structure the surface conductivity was found to exhibit ambipolar character, similarly to organic^13,14^ and inorganic^15,16^ semiconductors that exhibit electronic states within their band gap. The gradual increase of temperature has caused a corresponding loss of the ambipolar character of the surface conduction and of the sub-band light absorption, which vanished at 400 °C. The above were attributed to the oxidation of samples and to the improvement of atomic ordering, which in turn caused a decrease of the density of states (DOS) of the IB and of individual gap states. Upon cooling the samples down to room temperature the ambipolarity of conduction and the coloration reappeared. These phenomena were attributed to the reduction of samples upon cooling, which caused the re-creation of gap states and of the IB. Similar reversible phenomena, at lower however intensities, were observed during a second heating cycle performed on molybdenum oxide samples that had already subsisted a first thermal cycle.

## Experimental methods

Samples were deposited by hot-wire vapor deposition in a system described before^7^ composed of a stainless steel reactor in which the sample was positioned on an aluminum susceptor 2 cm below a molybdenum filament that was heated by an (AC) current lead by two Cu leads. The filament temperature was controlled by the current using calibration data obtained previously with a tiny thermocouple mechanically fixed on it. All depositions in this work were made with a filament temperature of 660 °C. Deposition time was used to control film thickness, which was near 90 nm with the substrate remaining at room temperature during deposition. The base pressure used was 80 mTorr, which was set using a commercial pressure stabilization system containing a diaphragm pressure gage (Baratron) and a PC-driven needle valve allowing for the flow of N_2_ through the reactor. From previous work it is known that as-deposited films are porous, amorphous and sub-stoichiometric in oxygen chemically described as MoO_2.7_^3,4,9^.

Silicon pieces, covered with a 100 nm thick SiO_2_ layer grown thermally, with dimensions of 2 × 2 cm^2^ cut from (100) Si wafers were used as substrates. Prior deposition the substrates were given a piranha clean^17^, washed in ultra-pure water and dried in a nitrogen stream. Samples destined for ellipsometric and simultaneous I–V measurements at various temperatures (see below) were prepared by depositing two square 1 × 1 mm^2^ Al plugs lithographically formed on top of the molybdenum oxide layer distant 2 mm from each other.

A specially designed accessory that allows for measurements up to 400 °C was adapted to a J.A Woolam Inc. M2000F rotating compensator ellipsometer. The ellipsometer was operating within the 250–1000 nm range running the WVASE32 software at an angle of incidence of 75.14°. The accessory was appropriately designed to operate without affecting the optical alignment of the optical beam, and moreover, it was allowing for I–V measurements simultaneously with the optical ones. For the I–V measurements two mechanically held tungsten probes were applied on the Al plugs formed on the sample, and a programmable KEITHLEY voltage source was used within the range −10 to 10 V with an increment of 0.1 V and a time delay, t_m_, of 10 ms between measurements (see also Fig. 3a). It is noted here that in the case of results reported in Figure S3 (see below), I–V curves were also recorded with increments of 0.1 V and time delays varying between 100 and 3000 ms.

SE and I–V measurements were made on a number of molybdenum oxide samples; results reported in this work are typical and were made on an as-deposited molybdenum oxide sample starting from room temperature up to 400 °C. The duration of heating was approximately 30 min and measurements were made every 10 °C. Then, the sample was left to cool down to room temperature with SE and I–V measurements made again in increments of 10 °C. The duration of cooling was 45–50 min. A second cycle of optical and electrical measurements followed on the same (now crystallized, see below) sample under similar conditions.

Molybdenum oxide samples were also deposited and characterized after thermal treatments at similar conditions of temperature and duration as for SE and I–V measurements. After deposition, samples were heated up to 400 °C with increments of 50 °C for a duration similar to that needed for a sample characterized by simultaneous optical and electrical measurements to reach this temperature. Then each sample was left to cool at room temperature and was characterized. The characterization included X-ray diffraction (XRD), Fourier transform infrared (FTIR) spectroscopy (in transmittance mode) and scanning electron microscopy (SEM) measurements.

For the XRD measurements a Philips goniometer using CuKa radiation and a graphite monochromator was used, for the FTIR a Bruker Tensor 27 spectrometer and for the SEM a LEO Supra 35 electronic microscope.

It must be noted that the condition of samples after the above thermal treatments does not correspond exactly to that of samples subsisting a continuous heating. However, valuable information can be obtained from their characterization as discussed in a next section.

## Results and discussion

### Amorphous molybdenum oxide films

Real and imaginary parts of the refractive index (referred to hereafter as the refractive index, RI, and extinction coefficient, EC, respectively) of molybdenum oxide films were obtained by fitting the experimentally recorded spectra with theoretical ones generated using a model that included three Lorentz oscillators. In all cases, the fitting was very satisfactory (see Fig. S1).

**Figure 1:**
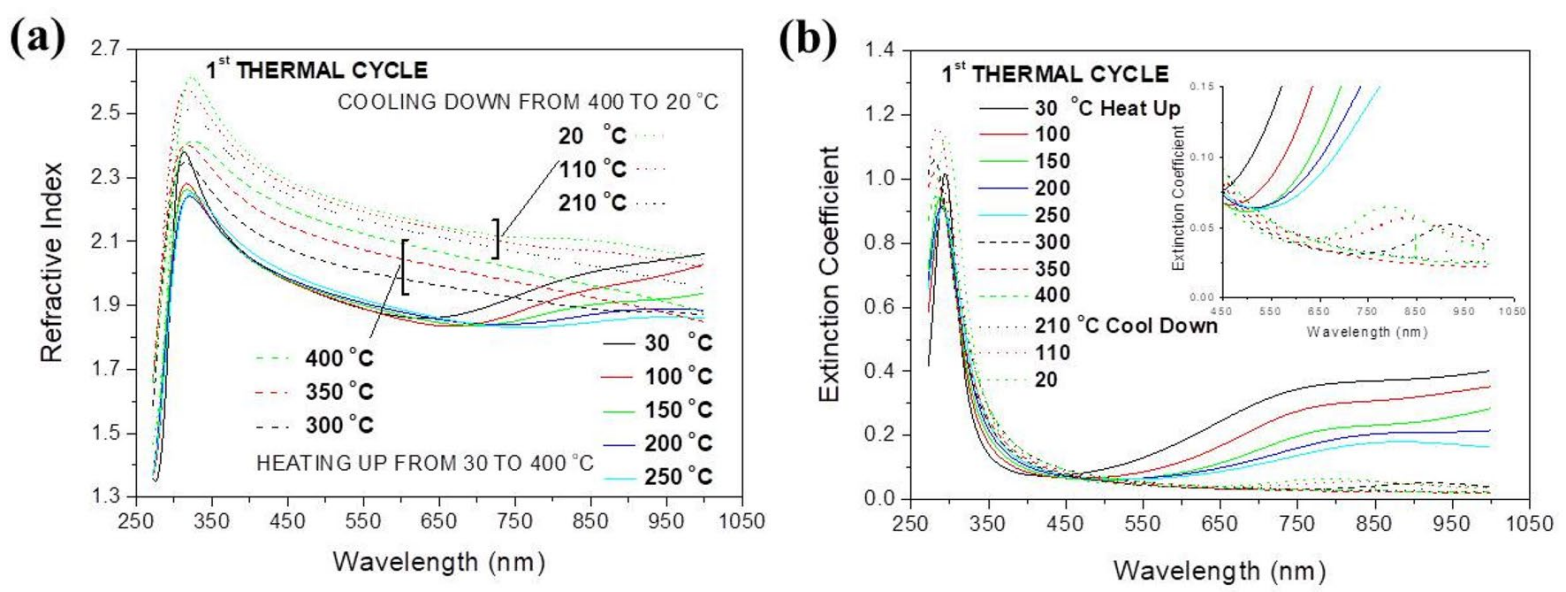
Temperature evolution of the dispersions of (**a**) the refractive index and (**b**) the extinction coefficient of an initially amorphous and sub-stoichiometric molybdenum oxide film during the first thermal cycle. The evolution of EC during heating above 300 °C and during cooling down to room temperature is shown at the insert of (**b**).

In Fig. 1 the temperature evolution of the RI (a) and EC (b) of a molybdenum oxide sample are shown. Maxima within the range of 250 to 350 nm in Fig. 1a, b correspond to the fundamental absorption while others, above 600 nm, to light absorption caused by electronic transitions between the IB and the CB, which cause the blue coloration of samples. As shown in previous works^17–19^, the bottom of the CB is composed mainly by 4d orbitals while the IB by unpaired 4d orbitals of Mo and its formation is related to oxygen sub-stoichiometry^9^. It is seen in the Fig. 1a, b, that with increasing temperature the sample oxidizes, and the atomic ordering improves (see below), the sub-band gap absorption decreases and at 400 °C it quenches completely (Fig. 1b). The sub-band gap absorption seen in Fig. 1 also extends within the infrared up to wavenumbers of (approximately) 2400 cm^−1^ (0.30 eV) as shown in Fig. S2, where FTIR transmittance spectra are shown taken within the range 4000 to 2400 cm^-1^ (2.5 to 4.16 μm) on heat-treated samples. The absorption decreases continuously with the heating temperature and vanishes completely at 400 °C similarly to the SE results shown in Fig. 1.

Returning to Fig. 1a, it can additionally be observed that the value of the RI within the range 350 to 650 nm increases with temperature from 1.8 initially, to approximately 2.1 at 400 °C indicating the densification of the film (discussed below) and increases further during cooling towards values corresponding to crystalline MoO_3_^20^. These observations indicate that the atomic re-arrangement in samples continues during cooling, i.e., the process except of temperature is also time dependent, as noted in the introduction. It is also observed that during cooling the sub-band absorption re-appears at 200 °C, as seen in the insert of Fig. 1b, and its intensity increases continuously as the temperature drops. However, at room temperature its intensity is lower than for the as-deposited film indicating that the population of gap states is now lower than for the same sample before heating.

**Figure 2:**
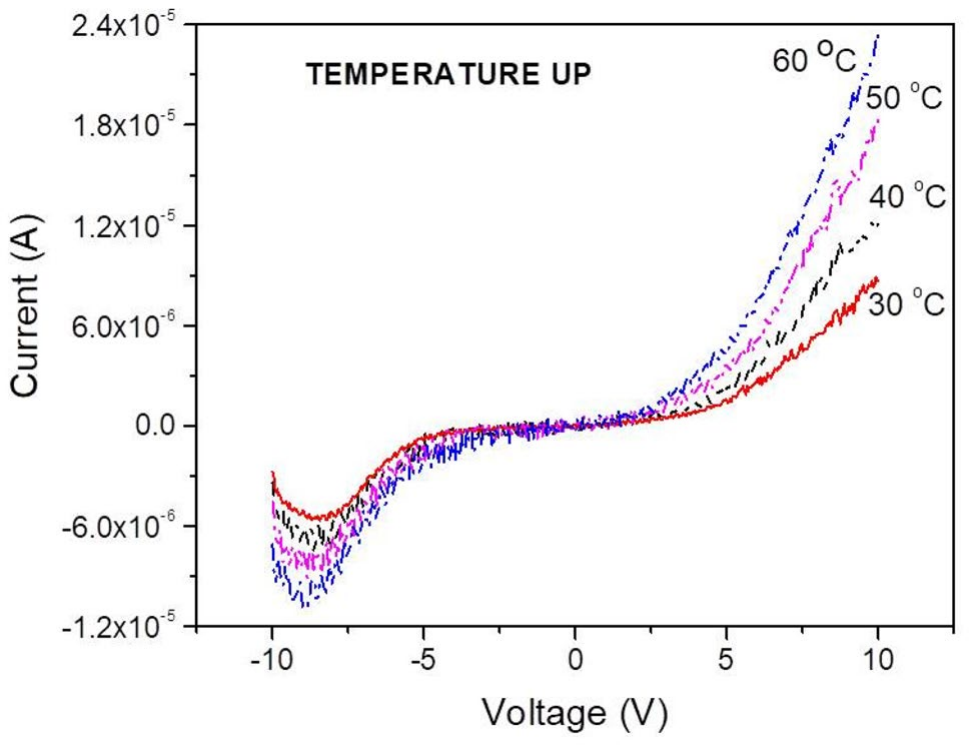
Current–Voltage characteristics taken on an Al/MoO_x_/Al sample at various temperatures within 30 and 60 °C.

The temperature evolution of the I–V curve for temperatures up to 60 °C is shown in Fig. 2. It is observed that, as the absolute value of the applied voltage decreases from −10 to 0 V the current initially increases and within the range of −8.5 to −9.0 V negative resistance is observed. After reaching a maximum, the current drops with the voltage, as intuitively expected. At positive voltages, a fast increase of current is observed after a turn-on voltage of 1.0 V.

**Figure 3:**
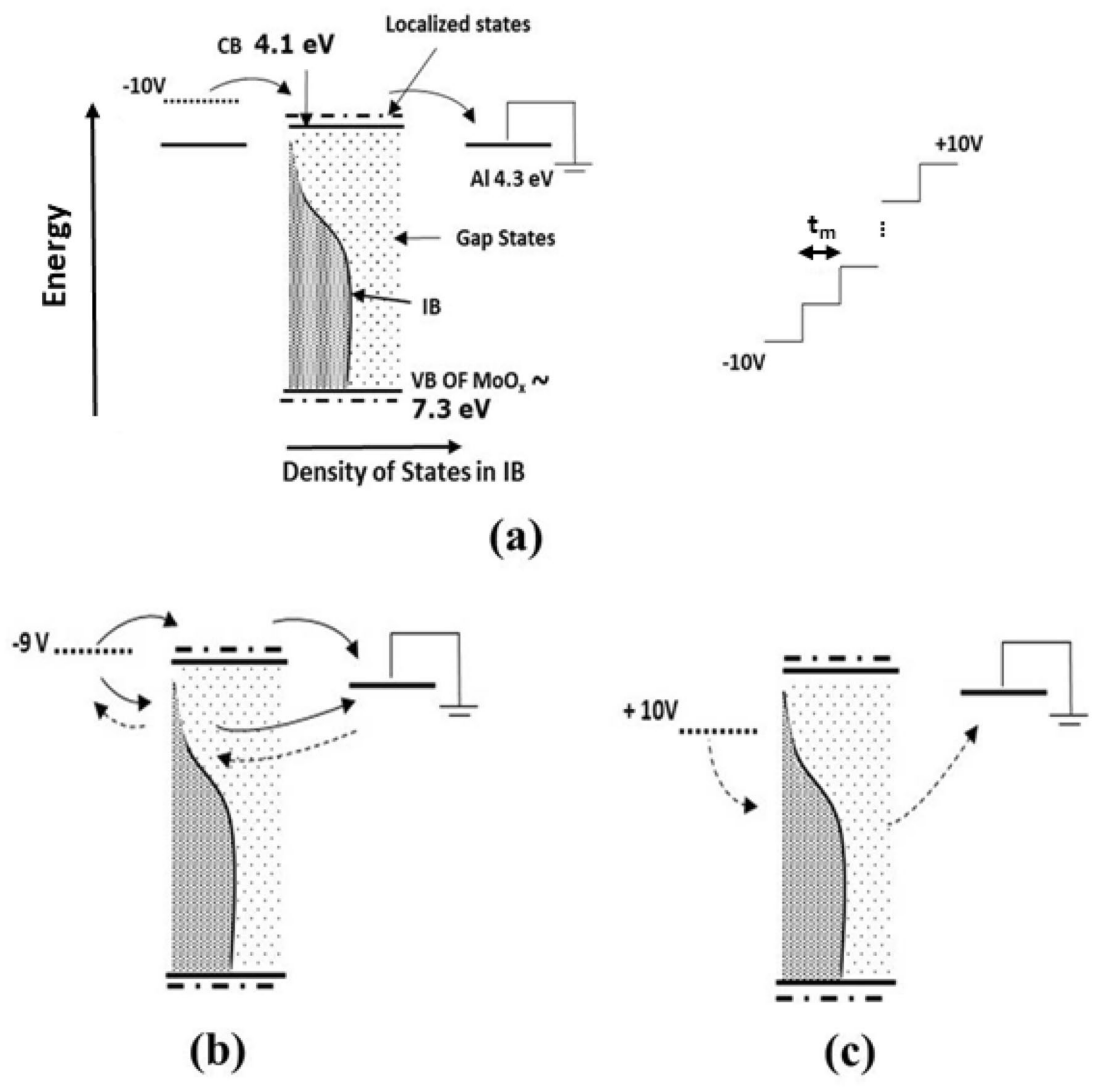
(**a**) The energy diagram of the Al/MoO_2.7_/Al structure (Left panel). The voltage ramp used for the I–V curve measurement is shown on the right. Each voltage is applied for a time delay t_m_. At voltages of −10 V the Fermi level of Al at the polarized contact (left) is shifted upwards and electrons (solid arrows) are injected in the CB composed by localized orbitals. As the voltage decreases, (**b**), the Fermi level approaches the top of the IB. Now except of electrons, also holes (dashed arrows) are attracted towards the electrode giving rise to ambipolar transport. (**c**) At positive voltages, the Fermi level is shifted near the centre of the IB, so only holes are injected.

Figure 3a shows the energy diagram of the Al/MoO_2.7_ (as-deposited)/Al sample based on earlier Ultraviolet Photoelectron Spectroscopy (UPS) measurements^3^. In the same figure is depicted the principle of the I–V measurements and the voltage ramp applied. The Fermi level of the Al electrodes lies at 4.3 eV^20^ and the CB of MoO_2.7_ at 4.1 eV. The low work function of the MoO_2.7_ is justified by the sub-stoichiometry in agreement with previous works^21,22^. The top of the VB is designed to be 3.2 eV (equal to the band gap^9^) below the bottom of the CB. The wave-functions at the top of the VB and bottom of the CB are strongly localized^12^ due to the disordered atomic arrangement in samples. Disorder also causes the creation of individual electronic gap states^12^. The IB is centred approximately 2 eV below the edge of the CB^9^ and exhibits an extended tail towards the CB in agreement with the data reported in Fig. S2. The sub-band gap absorption shown in Fig. 1b is caused by electronic transitions between the IB, the individual gap states and the CB, which is a dynamic process during which electrons are excited to the CB and holes are created in the IB.

When a negative voltage (−10 V) is applied on the left Al electrode, the Fermi level shifts upwards (see Fig. 3a), so electrons are injected in the bottom of the CB, which is composed by strongly localized orbitals. Therefore, their mobility is very low and they only move by thermal hoping, which is facilitated at high voltages only and this justifies the low current values observed in Fig. 2. With the applied voltage varying from −10 towards −9 V, the Fermi level shifts downwards, approaches the IB and injects electrons in it, shown with the continuous arrow in Fig. 3b. The process is facilitated by the large number of holes in the IB created during the process of sub-gap light absorption described above. This hypothesis was tested by recording I–V curves in dark and under room illumination. In Fig. S3 are shown typical I–V curves where it can be observed the enhancement of current caused by the illumination. Injected electrons and holes located near the Al electrode will neutralize and other holes will shift towards the electrode, so the whole process is equivalent to a current of holes shown with the dashed arrow in Fig. 3b, which adds to that of the electrons. It is seen then that the overall transport involves the displacement of electrons in the CB and of holes in the IB, i.e., it exhibits an ambipolar character. The participation of electrons and holes in the electrical conduction in MoO_3_ has been reported in the past by other workers, but the electronic structures of the particular materials in these works were not reported^23–25^. As a result of this kind of transfer, the overall current increases as the applied voltage drops, resulting in negative resistance as seen near −9 V in Fig. 2. It must be noted at this point that the 4d orbitals are considerably extended since their exponential part varies as e^-r/n^, where r is the distance from the parent nucleus and n is the principal quantum number (here equal to 4). The 4d electrons then may be found several interatomic distances away from the parent Mo ion, so they are easily attracted to (positive) electron vacancies, which is equivalent to a drift of holes. On the other hand, the overlapping of d-like orbitals is weak, so even a slight disorder may cause their localization^26^. It would be expected then that electrons at the bottom of the CB and holes in the IB exhibit comparable mobilities since both are composed by 4d orbitals. However, the 4d wave-functions of the IB, in agreement with the incertitude principle, are expected to be more extended than those at the CB because their energy is lower, so the mobility of holes is higher than that of electrons. The low mobility of the electrons at the bottom of the CB causes a negative charging of the film, which together with the high mobility of holes in the IB explains the suitability of amorphous and sub-stoichiometric MoO_x_ films for use as hole-transfer and electron-blocking layers in solar cells and light emitting diodes^3–8^. At positive voltages, the Fermi level shifts deep in the band gap, so the conduction becomes mainly due to holes, as seen in Fig. 3c. Electrons are also injected at the CB through the gap states, but, due to the strong localization of orbitals there, their contribution to the overall conduction is negligible. The barrier between the Fermi level of the polarized Al electrode and the center of the IB, which is of the order of 2 eV, as well as the localization of orbitals that compose this band justify the threshold voltage of + 1 V observed in the I–V characteristics of Fig. 2.

In order to investigate the role played by the localized states in the IB (and in the gap), I–V measurements were repeated within −10 to −7 V for various time delays (shown as t_m_ in Fig. 3a) and are reported in Fig. S4, where it is observed that the current increases with t_m_, as well as, the measurements noise. Moreover, the current maximum is shifted from −8.8 V at 100 ms to −9.5 V at 3000 ms. The above observations indicate that the current is due to charge carriers trapped at states in the gap and the IB, which are randomly attracted by the biased Al contact thus causing the observed increase of noise. At longer time delays energetically deeper traps have the time to empty causing the shift of the current maximum towards higher voltages.

**Figure 4:**
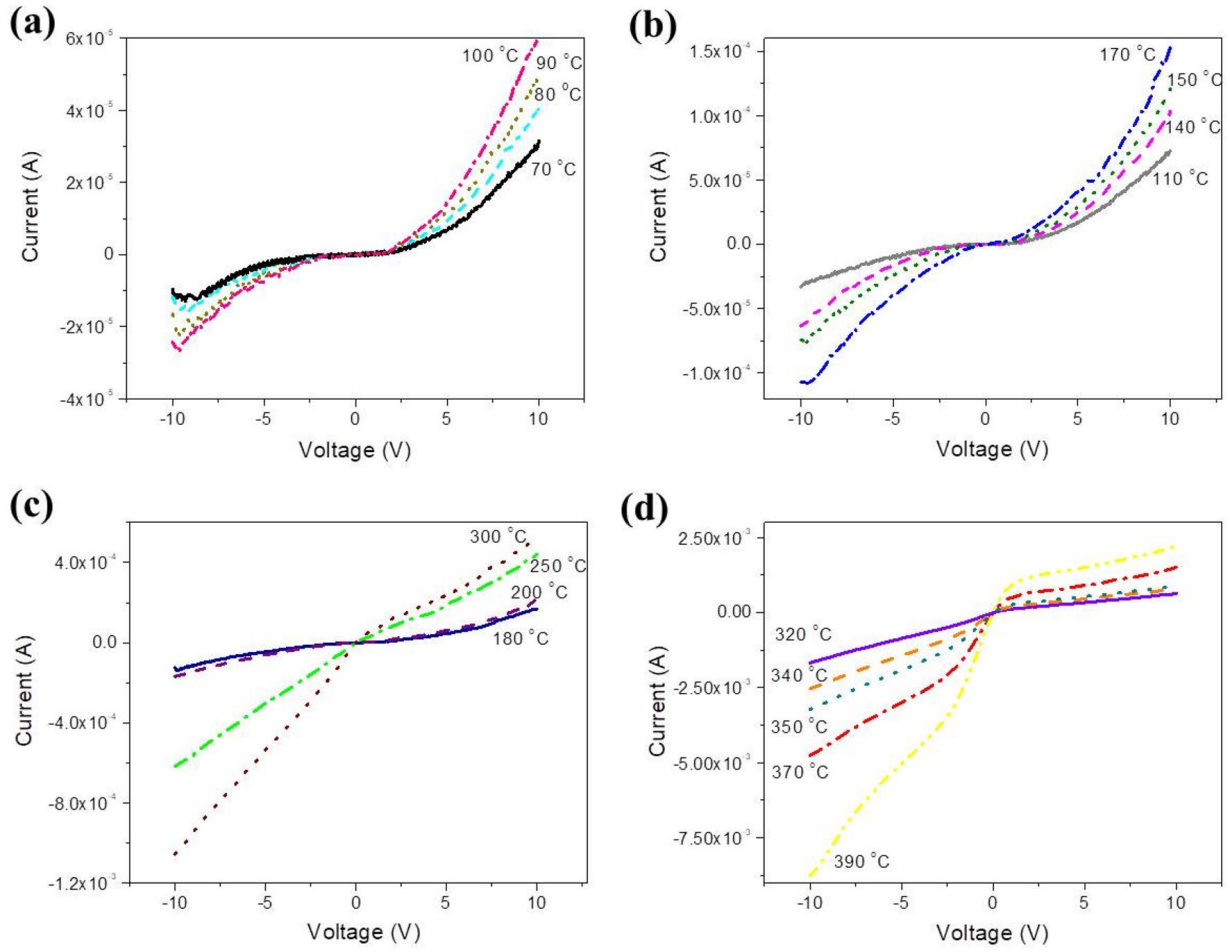
Temperature evolution of the I–V curve within the ranges (**a**) 70–100, (**b**) 110–170, (**c**) 180–300, and (**d**) 320–400 °C.

The temperature evolution of the I–V curve up to 400 °C is shown in Fig. 4a–d. It is observed that up to 100 °C (Fig. 4a), except of an increase of current due to the thermal excitation of carriers, the shape of the curve remains quantitatively similar to that for lower temperatures shown in Fig. 2. At temperatures within 110 and 170 °C (Fig. 4b) the I–V curve starts to become symmetric about the origin exhibiting, however, different slopes dependent on the polarity of the applied voltage and above 300 °C (Fig. 4c) currents at negative voltages exceed those at positive ones. At even higher temperatures (Fig. 4d), the I–V starts to resemble to that expected by a usual n-type semiconductor. At such high temperatures the atoms in films start to order periodically and this causes the delocalization of the wave-functions at the bottom of the CB and a significant increase of electron mobility. So, at negative voltages electrons injected in the CB, together with those thermally excited result in the high currents observed in Fig. 4d. At positive voltages, when the polarized junction is inversely polarized, only the leakage current is recorded due to holes thermally hopping through gap states. It must be noted at this point that devices similar to those in this work are been used in electronic gas sensing, where resistance changes are related to the composition of the chemical environment without to report the voltage at which measurements are made^27–29^. However, because of the non-linearity of the I–V curves, the voltage of measurement must be reported to facilitate the comparison between the various results. Before proceeding with the detailed discussion of the above observations, the temperature evolution of stoichiometry and atomic ordering will be discussed with the aid of a series of measurements including XRD, FTIR and SEM made on MoO_x_ samples treated at conditions (temperature and duration) similar to those as for the samples of Figs. 1, 2, and 4.

In Fig. S5, XRD curves taken on molybdenum oxide films treated at different temperatures are shown. As expected from previous work^3,4,9^ the as-deposited films are sub-stoichiometric in oxygen and highly disordered (porous, amorphous). The amorphous nature of samples is confirmed from the XRD spectrum in Fig. S5 where no peaks are observed (the peak near 70 degrees corresponds to the [001] crystallographic plane of the Si substrate).

To facilitate the description of the structure of amorphous MoOx a brief description of crystalline MoO_3_ is given based on Refs.^30,31^: The structure of this material is based on a series of bilayers each of which consisting of two sublayers joined with Van der Waals forces. Each sublayer is composed of distorted MoO_6_ octahedra held together by sharing the oxygen ions at their corners^30^ as seen in Fig. S6. This structure is very sensitive to oxygen stoichiometry; MoO_3_ can be reduced quite easily^30^ forcing octahedra to share edges, at low degrees of reduction and faces at higher degrees. There are at least six stable Magnéli phases^31^ leading to the lower stable oxide MoO_2_.

Amorphous and sub-stoichiometric MoOx, such as the as-deposited samples in this study, are composed by highly distorted MoO_6_ octahedra in which bond lengths and angles are distributed randomly, as shown in Fig. S7. In this structure the short range order (SRO) is low because Molybdenum and Oxygen ions are displaced relatively to the sites they occupy in the crystalline material and because many oxygen ions are lacking (x = 2, 7). As a result of this random distribution of ions unsaturated (dangling) bonds and voids are formed. Octahedra are still formed but now they are highly distorted and are joint to each other by sharing edges and faces randomly, so the long range order (LRO) is inexistent. This lack of LRO leads to the absence of Bragg reflections on the XRD spectrum of the as-deposited sample shown in Fig. S5 and of those taken on samples heated up to 250 °C. The morphology (Fig. S8a, b) and the FTIR spectra (Fig. S9) of these samples were not altered by heating relatively to the as-deposited one, thus corroborating further the lack of LRO up to this temperature. However, as seen in Figs. 1, 2 and 4a–c the electronic structure of these samples (from which optical and electrical properties arise) and their thickness (Fig. S10) were significantly influenced by heating. Heating in air is expected to cause the oxidation of samples, so it is seen how strongly the electronic structure of samples is influenced by oxidation, which causes the increase of Mo–O bonding sites, therefore improves the SRO and contracts the lattice parameters leading to the observed decrease of film thickness (Fig. S10).

The XRD spectrum for the sample heated at 300 °C exhibits peaks corresponding to various crystallographic planes of various sub-stoichiometric members of the Mo–O system^31^ indicating a significant improvement of LRO, which is also visible in Figs. S8c and S9. Further increase of temperature causes changes to the LRO manifested as new peaks on the XRD spectra corresponding to the Mo–O system and visualized on the SEM photos where large grains with dimensions of the order of μm are shown. The rhythm at which the film thickness contracts increases above 300 °C because the above described processes (named from here on “crystallization”) lead to a significant improvement of atomic packing and the formation of extended regions in films where the atomic ordering approaches that of single crystalline MoO_3_. As seen in Fig. S8e at 400 °C the laminated structure of grains is visible at the SEM images. The improvement of both, SRO and LRO in molybdenum oxide films during crystallization is also seen in the FTIR spectra reported in Fig. S9, where it is shown that up to 250 °C spectra exhibit smoothed features, while at higher temperatures sharp structures appear especially within the range 400 to 1000 cm^-1^, where the Mo–O bond vibrations absorb^32–35^. This process of crystallization continues during cooling adding to the thermal contraction a further shrinkage of thickness (Fig. S10). It is noted also that weak XRD peaks corresponding to stoichiometric MoO_3_ appear only on the spectrum of the film heated at 400 °C after it returned to room temperature. Of course the sample is not single-crystalline as seen from Fig. S8d–e where is shown that it is composed by grains with dimensions of μm in the bulk of which certainly exist point and extended defects, as dictated by thermodynamics. As seen in Fig. S8e the orientation of grains is random and their boundaries are expected to be composed by disordered material. The populations of defects in the bulk and the boundary are at dynamic equilibrium and during this process they combine and lead to a decrease of oxygen stoichiometry, as described in the introduction. The quantitative estimation (or measurement) of such small populations of defects and, consequently, oxygen sub-stoichiometry is impossible but their effect is clearly visible on the optical and electrical properties of samples as shown in Figs. 1, 2 and 4 and will be further discussed next.

**Figure 5:**
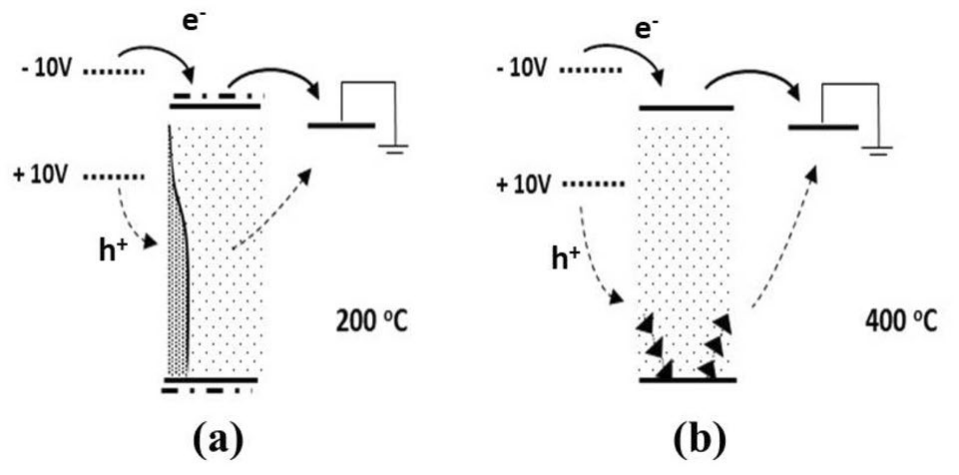
As the temperature increases above 200 °C the density of states of the IB decreases (**a**) and at 400 °C, when the sample is fully oxidized, it vanishes and, moreover, the wavefunctions at the bottom of the CB and the top of VB delocalize (**b**). A number of individual states remains within the gap giving rise to significant leakage current. (Note: The movement of electrons is represented by solid and that of holes by dashed arrows).

The I–V measurements of Fig. 4 may now be understood considering the thermal evolution of the electronic structure depicted in Fig. 5: At temperatures up to 100 °C the LRO has not changed but the oxidation has started, so SRO improved and this lead to a decrease of the DOS in the IB, therefore the discussion of the I–V shown in Fig. 4a is similar to that of Fig. 3. At higher temperatures, up to 200 °C (Fig. 4b, c), oxidation continues therefore SRO improves further while LRO remains almost unchanged. This has two effects: (i) the further decrease of the DOS in the IB as seen in Fig. 5a, and (ii) the decrease of the overall disorder in samples. So, a considerable number of states remains within the gap that facilitate the injection of both electrons and holes in the CB and the IB respectively and, additionally, the electrons mobility in the CB starts to improve because the wave-functions there start to de-localize due to the improvement of the LRO. Thus, the I–V curve becomes almost symmetric for negative and positive voltages as observed in Fig. 4c. Above 250 °C the current due to the injection of electrons in the CB becomes higher than that at positive ones which is due mostly to holes, because of the further de-localization of wave functions at the bottom of the CB. Moreover, the top of the IB quenches (Fig. 5a), holes now must overcome a barrier of the order of half the band gap in order to be injected in the IB, so the current at positive voltages is smaller than that for the electrons, as seen in Fig. 4c. At even higher temperatures the IB vanishes completely as seen in Fig. 5b, so the MoO_x_ (now x≈3) becomes similar to ordinary n-type semiconductors in which the electrons in the CB are donated by the individual states which still exist within the gap. At negative voltages electrons are injected easily at the CB by getting over a barrier of 0.2 eV thus resulting to high currents (see Fig. 4d), which increase further because of the thermal excitation of carriers at gap states. At positive voltages the MoO_x_/Al (right) contact is inversely polarized, so only the leakage current is monitored due to holes emitted from the polarized contact by thermal hoping through individual gap states as seen in Fig. 5b.

Another point that must be discussed is the temperature evolution of the work function of the molybdenum oxide and of its interface with Al. Figures 3 and 5 have been designed based on previous UPS measurements^3^ in which the work function of MoO_2.7_ was found 5.9 eV, much lower than that for stoichiometric molybdenum oxide, which is known to be near 7 eV^19,30^. This reduced value was attributed to the oxygen sub-stoichiometry in agreement with results reported in^21^. Oxidation is expected to cause an important increase of the oxide’s work function and if that of the interfaces remains unaffected, it would result to a shift of both Al/oxide interfaces deep into the CB of the oxide (see Fig. S11). In such a case, the conduction would be due exclusively to electrons, which at negative voltages would have to overcome a barrier of approximately 3 eV to reach the grounded electrode resulting in low currents at negative and in high currents at positive voltages, contrary to results reported before. A possible explanation is that the oxidation also causes a shift of the interface Al/oxide towards higher energies leaving the overall energy structure almost unaffected. Such a phenomenon has been reported in the past^22^ for the interface of molybdenum oxide with various metals, so a similar behaviour is expected for Al.

**Figure 6:**
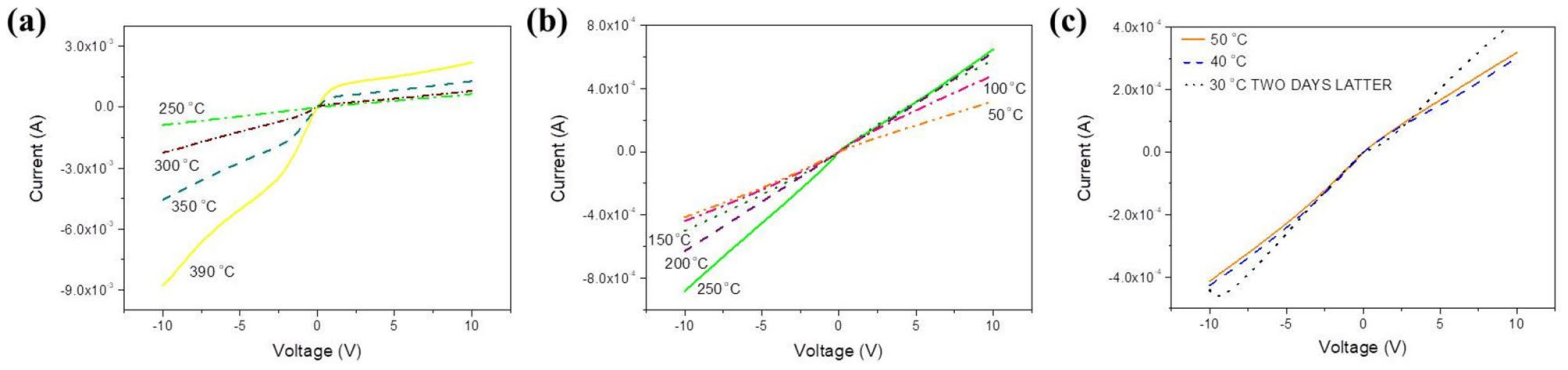
Temperature evolution of the I–V curve within the ranges (**a**) 400–250, (**b**) 250–100, and (**c**) 40, 50 °C and at room temperature two days after measurement. It is observed in (**c**) that as the temperature decreases the current at negative voltages increases contrary to what is expected for a semiconductor.

The I–V curves recorded during cooling are shown in Fig. 6. It can be observed that cooling has caused the inverse evolution of the I–V curves than heating but since SRO and LRO are now considerably enhanced relatively to the as-deposited ample, the mobilities of electrons and holes are now higher and consequently the currents recorded are enhanced relatively to those in Figs. 2 and 4. Down to temperatures of approximately 200 °C a small number of states exist in the gap (see Fig. 5), so the I–V curve is that of a typical metal/n-type semiconductor/metal structure, in which one junction is forward and the other inversely polarized. As the temperature decrease the number of oxygen vacancies starts to increase as described in the introduction and their random distribution promotes the formation of states in the gap, which facilitates the movement of holes, so the I–V curve becomes symmetric again about the origin (Fig. 6b). Further decrease of temperature (below 200 °C) causes a further increase of the number of gap states and the re-formation of the IB, which can be verified by the re-appearance of the sub-bandgap absorption band observed in Fig. 1a, b. Now, as seen in Fig. 6c, the current at negative voltages, caused by the collective contribution of electrons and holes drifting in the CB and the IB respectively is higher than that at positive voltages, which is due to holes only. An interesting feature that must be pointed out here is that at 40 °C and negative voltages the current recorded is higher than that at 50 °C, which is an unexpected result for a semiconductor. This can be explained by the collective participation of electrons and holes in the overall electric conduction at negative voltages, i.e., the ambipolar character of the electric transport in MoOx films (see also below).

As seen in Fig. 6c the current has increased further after two days. This can be attributed to the continuous creation of oxygen vacancies and consequently the further reduction of samples at room temperature during this time interval. As discussed before, at the end of the first heating cycle the material was ordered (see Figs. S5, S8, S9), so the creation of oxygen vacancies and consequently its reduction did not influence SRO and LRO but only caused the descent of unpaired 4d orbitals from the CB to the IB, i.e., the increase of the DOS in it. Consequently, the width of the IB has also increased, which in turn caused a decrease of the effective mass of holes and the increase of their mobility. It is seen then that the assumption that at room temperature the sub-stoichiometry is thermodynamically favoured in molybdenum oxide films, as discussed in the introduction is justified, since allows for a coherent discussion of results obtained by electrical and optical measurements.

### Crystallized molybdenum oxide films

In order to further investigate the role of atomic ordering and oxygen stoichiometry on the electric transport in molybdenum oxide films, a second cycle of electrical and optical measurements with varying temperature was made on samples having already undergo a first heating cycle at the end of which, as seen before, their atomic ordering has improved substantially relatively to the as-deposited samples (hereafter referred to as crystallized samples).

**Figure 7:**
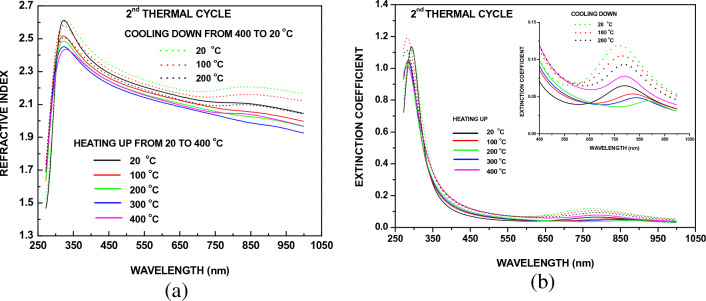
Temperature evolution of the real part of (**a**) refractive index and (**b**) extinction coefficient of a molybdenum oxide sample during the second thermal cycle.

In Fig. 7a, b the real and imaginary parts of refractive index of the crystallized molybdenum oxide sample are reported. The sample exhibits sub-band gap absorption but with significantly lower intensity relatively to the as-deposited samples (Fig. 1a, b). As the temperature of measurement increases the sub-gap absorption band decreases but contrary to the as-deposited samples, does not vanish even for temperatures as high as 400 °C where a weak absorption band is still observed (Fig. 7b) indicating that a significant number of states remains in the gap at this temperature. Similarly to the first thermal cycle, as the temperature decreases the sub-gap absorption increases as seen at the insert of Fig. 7b, indicating that the number of gap states also increases. And since the origin of gap states is the oxygen sub-stoichiometry and the atomic disorder this observation corroborates the conclusion drawn before that at room temperature reduced molybdenum oxide films are thermodynamically more stable than the fully oxidized ones also when they are crystallized.

**Figure 8:**
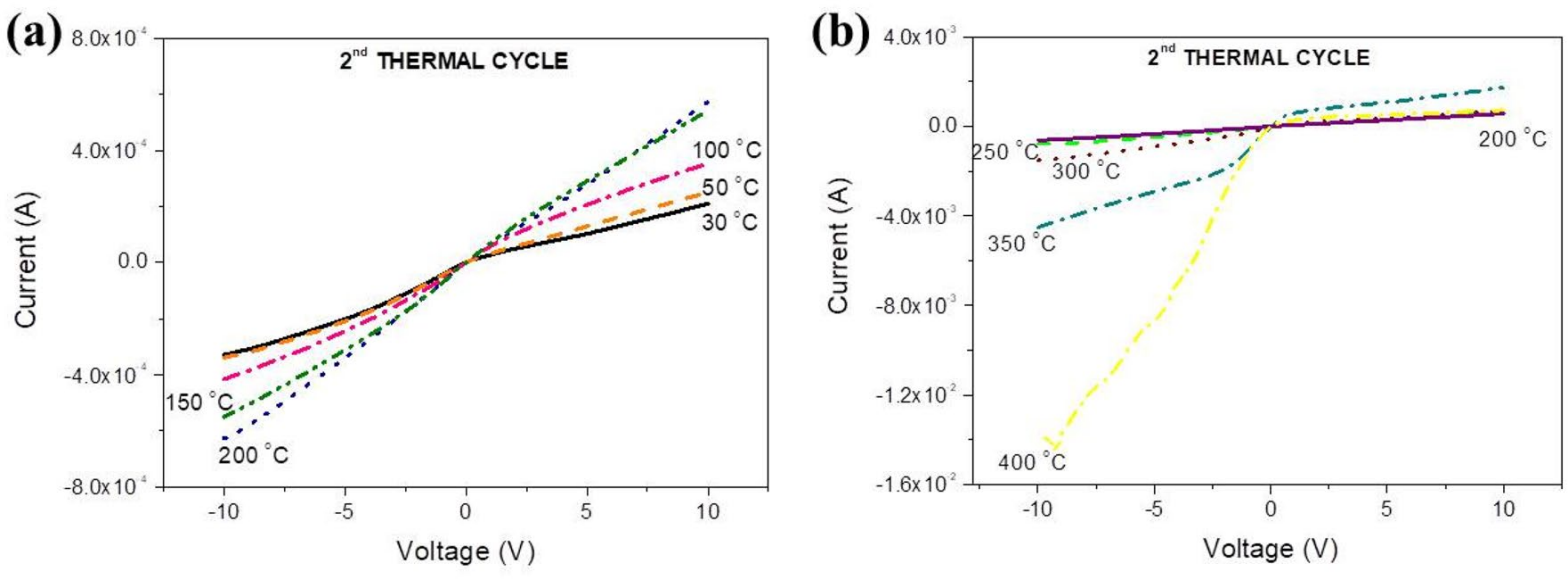
Evolution of the I–V curve during the second thermal cycle while heating up within the ranges (**a**) 30–200 and (**b**) 200–400 °C.

**Figure 9:**
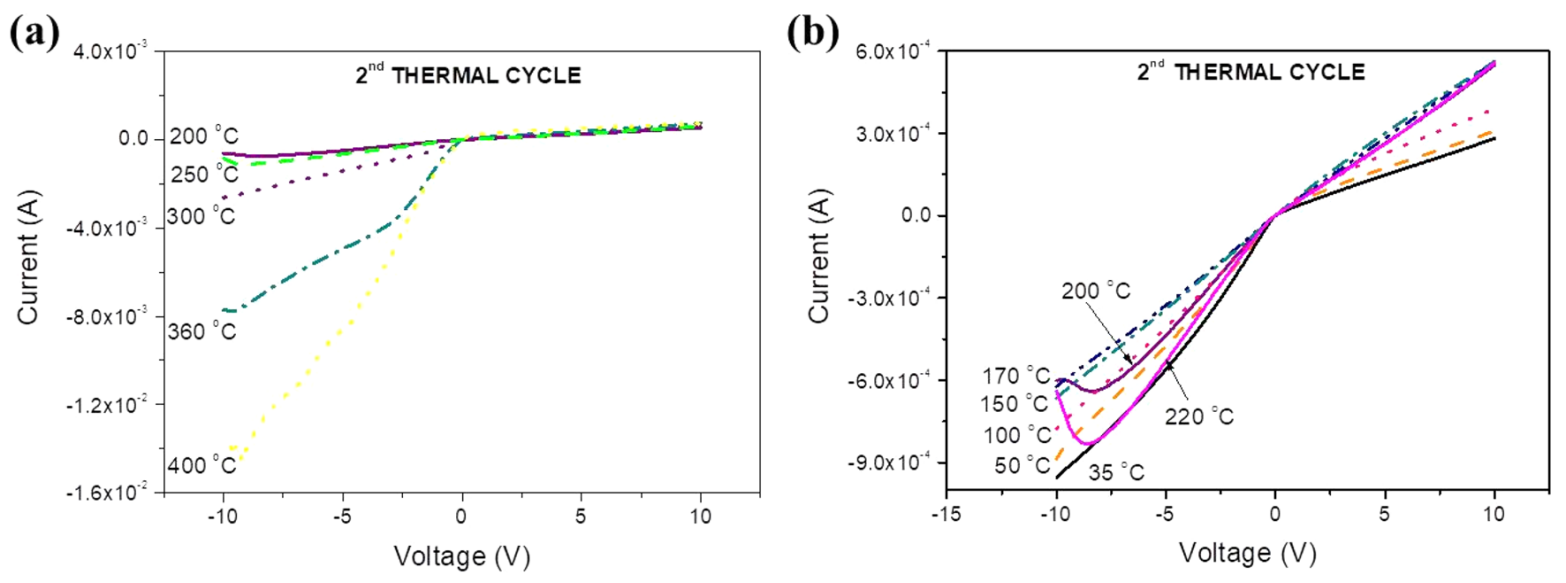
Evolution of the I–V curve during the second thermal cycle during cooling down from (**a**) 400 to 200 °C and (**b**) 200 to 35 °C. A “metallic” behavior is observed at negative voltages, where the current increases as the temperature drops from 170 to 35 °C.

The temperature evolution of the I–V curve of crystallized samples during heating up is shown in Fig. 8a, b and can be interpreted in terms of the electronic diagrams of Figs. 3 and 5 taking into account that now the bottom of the CB and the IB are composed by extended wave-functions, so electrons and holes there exhibit higher mobilities than for the amorphous material. At temperatures up to 100 °C, due to the oxygen sub-stoichiometry a considerable number of states exist within the gap and the IB is present, so at negative voltages the overall current through the sample is due to electrons injected in the CB and to holes moving through the IB towards the polarized electrode. This ambipolar transport explains the higher currents measured at negative voltages relatively to the positive ones where the current is due to holes injected in the IB only. Within the range 100–300 °C the curve becomes symmetric because the DOS in the IB and the number of gap states decrease significantly. Above 350 °C (Fig. 8b) the I–V becomes similar to that of an ordinary n-type semiconductor because the CB is composed by extended orbitals, so at negative voltages higher currents than during the first cycle are observed and the leakage current is lower than for the first thermal cycle because of the significant decrease of the overall number of gap states where holes move. It is also observed that, due to the existence of the IB at 400 °C a voltage domain of negative resistance is still observed near −10 V (Fig. 8b). Such domain does not exist at temperatures up to 350 °C probably because the DOS in the IB is low as seen in Fig. 7b where the sub-gap light absorption at 400 °C is much higher than at lower temperatures. Α possible explanation for this effect is that as the temperature raises, at 350 °C there is not enough time for a “dense” IB, containing numerous orbitals to form but it has enough time to form later when the temperature reaches 400 °C. This suggestion is corroborated by the observation that during cooling the domain of negative resistance is present at 360 °C, as seen in Fig. 9a. It must be noted that the formation of gap states and of the IB is a result of many processes: formation of point and extended defects, oxygen diffusion, atomic packing and ordering, etc. All these processes are time-dependent and thermally activated with activation energies that vary with the temperature, the oxidation state, the structure and the morphology of the film, etc. A re-adjustment of all these activation energies combined with the time-varying character of these processes provide a possible explanation for the IB to form at several temperatures (and times) and not to others.

The evolution of the I–V curves during cooling is reported in Fig. 9a, b. It is observed that at 400 and 350 °C the region of negative resistance is shown near −10 V, disappears at 300 °C and re-appears within the range 250 to 200 °C. These phenomena are probably related to the exponential variation and the time dependence of the involved processes, as discussed above. Below 200 °C a curious effect is observed as seen in Fig. 9b. At negative voltages the current drops with temperature down to 170 °C, as expected for a semiconductor, and for lower temperatures it starts to increase again. A similar phenomenon of “metallic” temperature variation of current when electrons are injected was observed during the first cycle (Fig. 6c). Also, below 170 °C ranges of negative resistance are no longer observed because as seen in Fig. 7b, during sample cooling the sub-gap absorption band gets stronger and its width increases to cover practically the entire gap. So, as the temperature drops the DOS in the IB increases as well as the width of this band, therefore the effective mass of holes there gets smaller and consequently their mobility increases. Due to the enhanced LRO the wave functions at the bottom of the CB and in the IB become extended. So, at negative biases electrons and holes at the bottom of the CB and in the IB respectively participate in the overall current simply by drifting and not by thermal hopping between localized states as during the first thermal cycle, which explains the lack of ranges with negative resistance.

## Summary and conclusions

An accessory was designed and adapted to a commercial ellipsometer, which allows for I–V measurements simultaneously with the optical ones at temperatures up to 400 °C. Such measurements were made on samples of the kind: Al/MoO_x_/Al (x ≤ 3) and the electric transport in them was related to the electronic structure of the oxide that depends on oxygen stoichiometry and atomic ordering, both of which changing with temperature.

The conclusions drawn from this study are: Amorphous and crystallized molybdenum oxide layers at room temperature and in ambient air are sub-stoichiometric in oxygen. The oxygen sub-stoichiometry induces the emergence of an intermediate band (IB), the structural disorder causes the appearance of individual electronic states within the band gap and cause the localization of the wave-functions that compose the top of the VB, the bottom of the CB and the IB. At near room temperatures, electronic transitions between the IB and the conduction band cause additional light absorption at photon energies below the energy of the gap, resulting in the blue coloration of films. In amorphous MoO_x_ films, when electrons are injected in the CB by applying a negative voltage to one of the two junctions, holes moving in the IB are also attracted towards it, so the total current is due to both kinds of carriers, therefore the transport is characterized as ambipolar. At temperatures up to 100 °C the mobility of holes is comparable to that of conduction electrons because of the strong localization of wave-functions that compose the bottom of the CB. As the temperature increases samples oxidize and the atomic ordering improves, the IB gradually attenuates and the number of gap states decreases, so the transport loses gradually its ambipolar character. In initially amorphous molybdenum oxide samples the IB quenches completely at 400 °C and at this temperature the transport becomes similar to that of usual n-type semiconductors. Cooling samples back to room temperature causes their reduction, so IB and gap states form again and therefore the blue coloration, as well as the ambipolar character of the conduction re-appear. Crystallized molybdenum oxide samples, contrary to the amorphous ones, do not oxidize completely even at temperatures as high as 400 °C and reduce when cooled back to room temperature. In such samples the IB remains at all temperatures together with the ambipolar transport, giving rise to a strange phenomenon of electron currents that increase instead of decreasing with temperature, which is not a behavior expected by a semiconductor. This phenomenon is due to the formation of the IB and of gap states that form as the temperature drops and the molybdenum oxide samples reduce.

### Supplementary Information

Supplementary Information.

## Data Availability

The datasets used and/or analysed during the current study available from the corresponding author on reasonable request.
