# Supplementary material for: Ambipolar surface conduction in oxygen sub-stoichiometric molybdenum oxide films
Source: Sci Rep. 2023 Nov 30;13:21166. doi: 10.1038/s41598-023-48060-1 (PMC10689454; doi:10.1038/s41598-023-48060-1)
Supplement: Supplementary file 1 — Supplementary Information. [file 41598_2023_48060_MOESM1_ESM.docx]

**Supporting Information**

**Ambipolar surface conduction in oxygen sub-stoichiometric molybdenum oxide films**

Anastasia Soultati^1^, Konstantinos Aidinis^2,3^, Alexander Chroneos^4,5,*^, Maria Vasilopoulou^1,*^, and Dimitris Davazoglou^1,*^

*^1^NCSR “Demokritos”, Institute of Nanoscience and Nanotechnology, POB 60228, 153 10 Agia Paraskevi, Attiki, Greece.*

*^2^Department of Electrical and Computer Engineering, Ajman University, P.O. Box 346, Ajman, United Arab Emirates.*

^3^Center of Medical and Bio-allied Health Sciences Research, Ajman, United Arab Emirates.

*^4^Department of Electrical and Computer Engineering, University of Thessaly, 38221 Volos, Greece.*

*^5^Department of Materials, Imperial College, London SW7 2AZ, United Kingdom.*

|  |  |
| --- | --- |
| **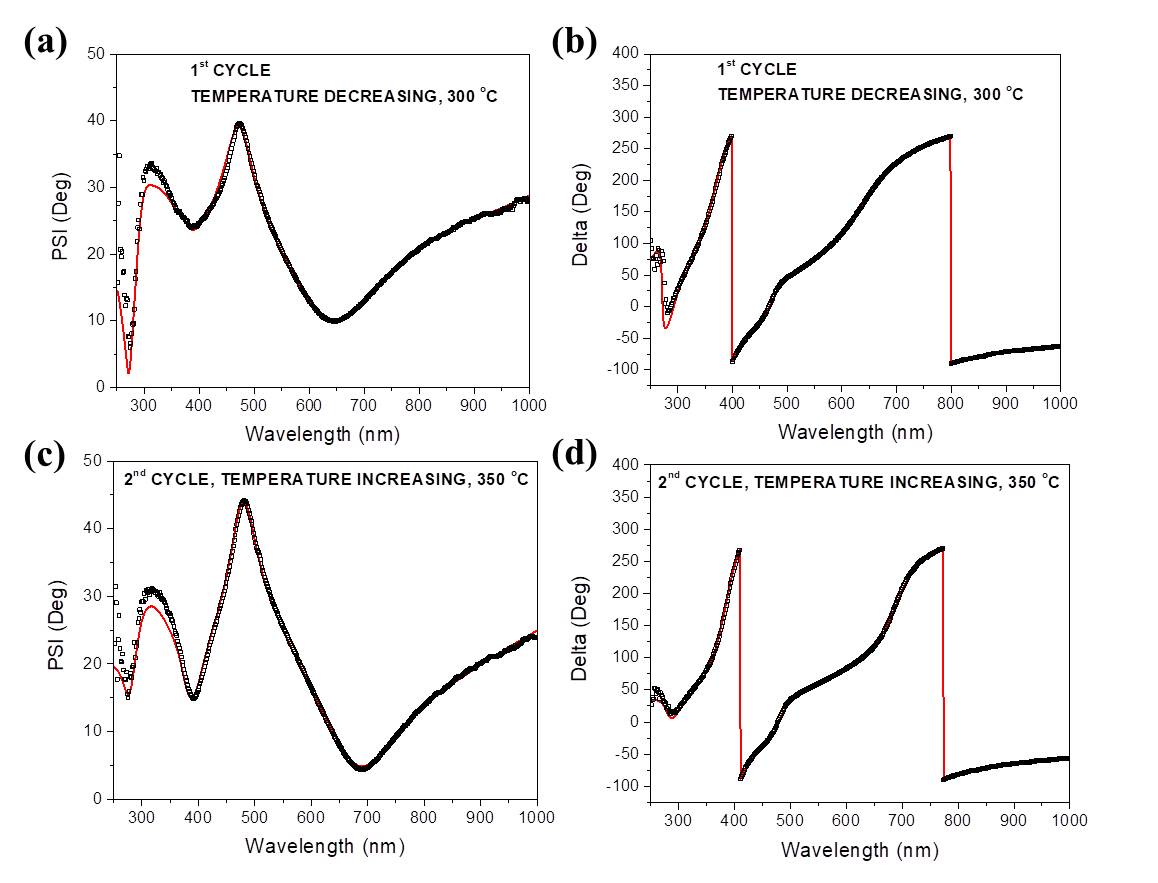**  **Figure S1**. Typical experimentally recorded (black) and theoretical (red line) spectra of a molybdenum oxide sample during the first thermal cycle while cooling down from 300 ^o^C ((a) and (b)) and up to 350 ^o^C ((c) and (d)). The theoretical spectra of the ellipsometric constants, (a), (c) Psi and (b), (d) Delta were generated using a three Lorentz oscillator model. | |

|  |  |
| --- | --- |
|  | |

**Figure S2**. FTIR spectra taken on molybdenum oxide samples heated at temperatures up to 400 ^o^C. The absorption band centred at 3400 cm^-1^, which is the low-energy side of the band seen in Fig. 1(b) extends in the infrared up to wavenumbers of 2600 cm^-1^ (0.32 eV) and decreases continuously with heating to vanish at 400 ^o^C. The observed absorption bands are due to electronic transitions between states located within the gap and the conduction band.

**Figure S3.** Typical I-V characteristics recorded on an Al/MoOx/Al sample in dark and under room illumination at 25 ^o^C.

**Figure S4**. Current-Voltage characteristics taken on an Al/MoO_x_/Al sample at room temperature from -10 to -7 V for time delays between 100 (upper) and 3000 ms (lower curve).

**Figure S5**. XRD spectra taken on amorphous as-deposited molybdenum oxide films heat treated at various temperatures in air. Peaks, corresponding to crystalline forms of molybdenum oxide start to appear above 300 ^o^C. The peak near 70 degrees is due to the silicon substrate.


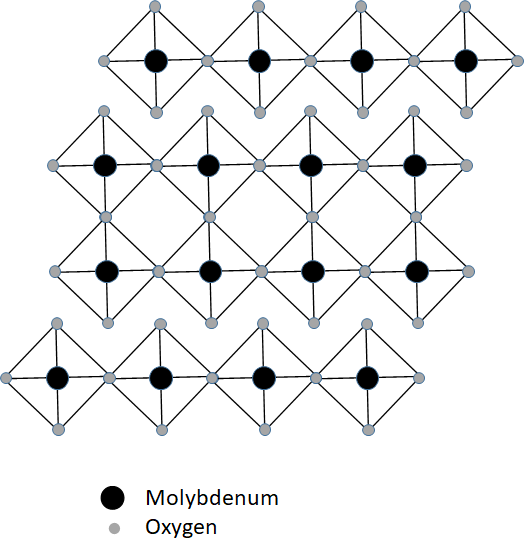


**Figure S6.** Schematic representation of the structure of crystalline MoO_3_. The double-layers are shown that are composed by MoO_6_ oxtahedra bonded by sharing the corner oxygen ions. Double layers are held together with van-der Waals forces.


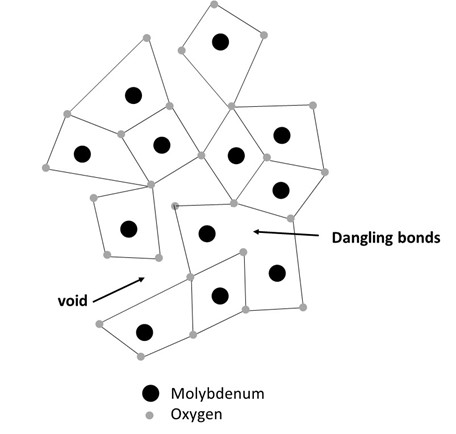


**Figure S7.** Schematic representation of the amorphous structure of MoOx. The structure is sub-stoichiometric in oxygen because the MoO_6_ octahedra (when formed) are bonded by sharing edges and faces. Voids and dangling bonds are shown.

| **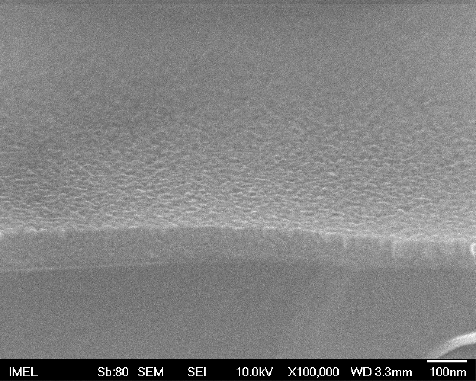**  **(a)** | **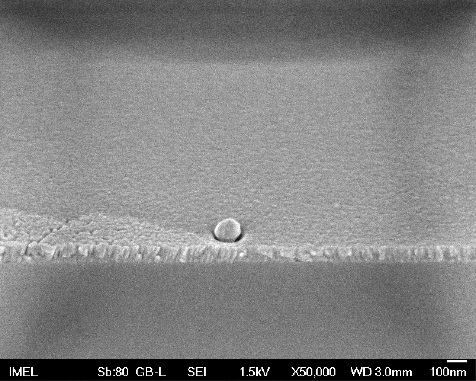**  **(b)** |
| --- | --- |
| **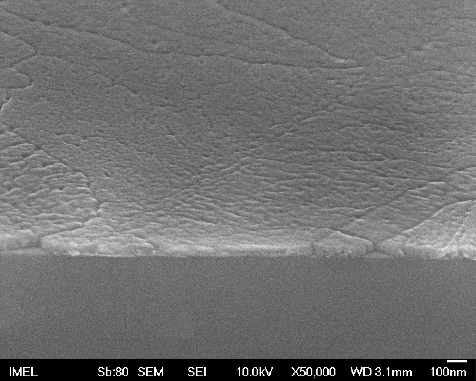**  **(c)** | **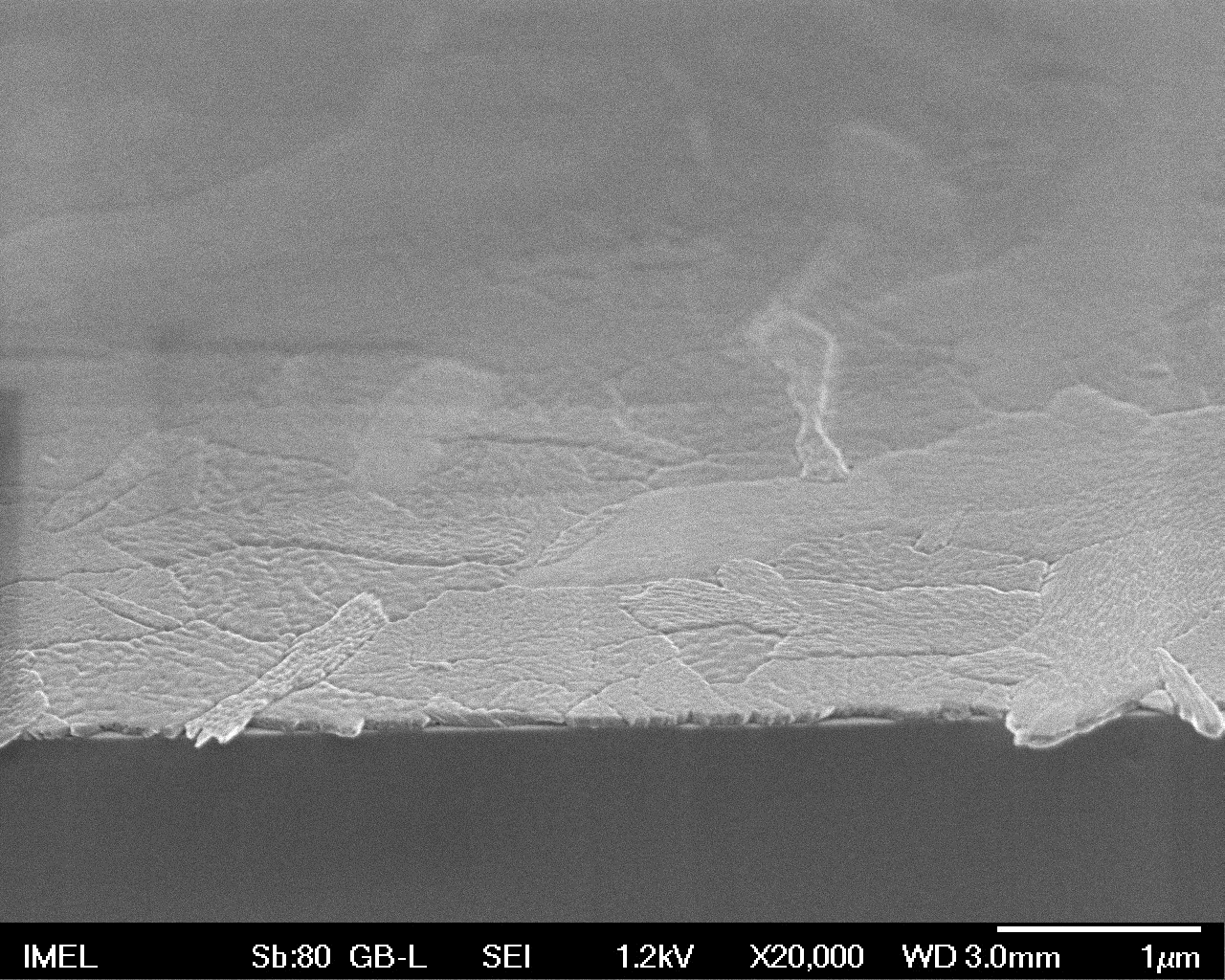**  **(d)** |
| **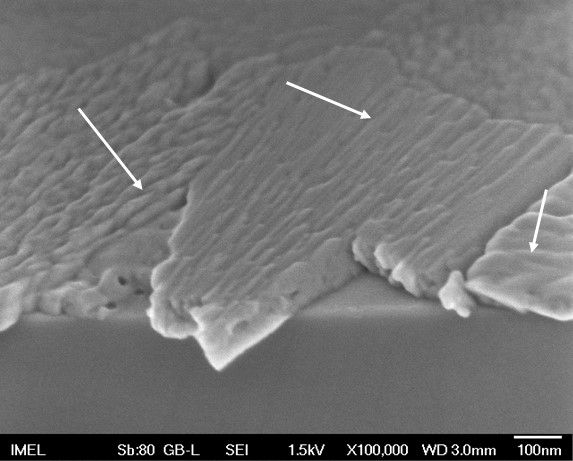**  **(e)** | |

**Figure S8.** SEM photos taken on the surface of four different samples treated at: (a) 70, (b) 250, (c) 300 and (d) 400 ^o^C. Up to 250 ^o^C films morphology does not change relatively to the as deposited films, while above this temperature large grains are formed whose size increases with temperature (note the scale change for micrograph (d)). The lamellar structure of the grains formed above 350 ^o^C is indicated with the arrows in (e)

**Figure S9**. FTIR spectra taken on the same as in Fig.’s 2 and 5 samples. Structures, indicating enhanced LRO start to appear at 250-300 ^o^C. ~~The observed absorption bands are due to electronic transitions between states located within the gap and the conduction band.~~

**Figure S10**. Temperature evolution of the thickness of the molybdenum oxide sample during the two thermal cycles.


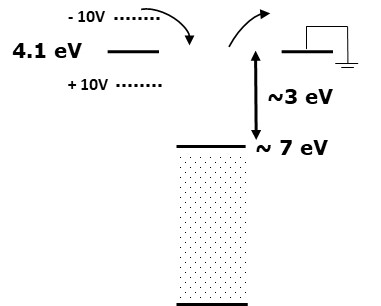


**Figure S11**. If during heating the work function of molybdenum oxide increased up to 7 and that of Al remained at 4.1 eV, only electrons through the CB, would participated in transport and had to overcome potential barriers of the order of 3 eV.
